# Active Video Games and Low-Cost Virtual Reality: An Ideal Therapeutic Modality for Children With Physical Disabilities During a Global Pandemic

**DOI:** 10.3389/fneur.2020.601898

**Published:** 2020-12-14

**Authors:** Marika Demers, Ophélie Martinie, Carolee Winstein, Maxime T. Robert

**Affiliations:** ^1^Division of Biokinesiology and Physical Therapy, University of Southern California, Los Angeles, CA, United States; ^2^Center for Interdisciplinary Research in Rehabilitation and Social Integration, Quebec City, QC, Canada; ^3^Department of Rehabilitation, Laval University, Quebec City, QC, Canada

**Keywords:** COVID-19, active video game, virtual rehabilitation, cerebral palsy, telerehabilitation

## Abstract

Current guidelines against spread of coronavirus (COVID-19) interrupt non-essential rehabilitation services. Thus, individuals with physical disabilities such as children with cerebral palsy can no longer benefit from physical rehabilitation during this undetermined period. Using either a synchronous or asynchronous format, in collaboration with a therapist via telerehabilitation, we suggest that active video games and low-cost virtual reality are a promising delivery mode for at-home rehabilitation in the context of a global pandemic. This therapeutic modality, incorporated into an at-home individualized treatment plan, provides a means to lessen the impact of an interruption in rehabilitation services while not loosing the pre-pandemic, in-person physical activity gains. Growing evidence supports active video games and low-cost virtual reality as viable therapeutic interventions for children with physical disabilities. These technologies are especially well-accepted by pediatric populations for the ludic and motivating features that lend themselves to nearly seamless incorporation into telerehabilitation. Advantages for rehabilitation of active video games and low-cost virtual reality include a rich, challenging, multi-modal training environment in which high numbers of movement repetitions can be accomplished, and a unique opportunity to foster engaged practice actions that go beyond household activities. We offer suggestions for the clinician about how to adopt active video games and low-cost virtual reality into your practice during a global pandemic.

## Introduction

Current strategies to combat the Coronavirus (COVID-19) involve flattening the incidence curve and reducing the rate of spread, such as social distancing, school closures, and confinement of the population (1–3). While these preventives measure effectively reduce the rate of COVID-19 on a population scale, individuals with physical disabilities and their families are facing unique challenges, including disruption of daily routines, and limited access to rehabilitation programs (4). The World Confederation for Physical Therapy among several other professional associations worldwide recommends to either stop or post-pone rehabilitation services that are considered non-essential (5). In a recent survey of health professionals across 35 European countries (6), rehabilitation outpatients' activities were stopped in 83% of countries, with an estimated range of 1.3–2.2 million Europeans deprived of rehabilitation services. The negative consequences of such an interruption is especially high for children with physical disabilities; a vulnerable population for which these critical services bring access to valuable resources that are essential to maintain physical abilities during natural development and prevent secondary complications. There are some important parallels to the global trends in aging and the increased prevalence of multiple chronic conditions that sparked development of a subfield in rehabilitation science at the nexus of new technologies, aging and disability (7). This perspective focuses directly on the impact of the COVID-19 response for children with physical disabilities. We propose that in-home therapy which enlists engaging technologies such as active video gaming and low-cost virtual reality, offers a promising solution to minimize the impact of long-term interruptions in rehabilitation services. Active video gaming is defined as video games that require interactive physical activity beyond being controlled passively through conventional hand controller (8). Virtual reality in the context of a rehabilitation program is described as an intervention delivered through virtual reality platforms that involves real-time simulation of an engaging environment and allows the user to interact via multiple sensory channels (9). These possible therapeutic modalities may be an ideal means to maintain and even advance gains achieved through in-person therapy and thereby prevent further functional decline likely to ensue as a consequence of interruptions in rehabilitation services.

Among children with physical disabilities typically followed by rehabilitation specialists, cerebral palsy (CP) is the most common neuromotor disorder in children (10) with a prevalence ranging from 1.5 to 2.5 per 1,000 live births (11–13) and an estimated lifetime cost of 1 million dollars (14). Children with CP and other pediatric populations with physical disabilities have a wide range of impairments, including muscle tone disorders (15), reduced sensation (16, 17), reduced aerobic capacities (18), and cognitive deficits (19, 20). Ultimately, these physical and mental impairments negatively affect most activities of daily living; this inevitably leads to reduced levels of participation in both leisure and physical activities (21, 22). Unfortunately, sensorimotor impairments very often perpetuate a lifetime cycle of degeneration of health status (18).

Aside from sensorimotor impairments, limited participation is attributed to a number of obstacles including: personal, socioeconomic status and environmental factors (23). Other hindrances include lack of community programs that cater to children with disabilities (24, 25), limited resources to adapt the environment (26) and lack of accessibility to resources for the parents (27). In the current global pandemic, confinement can accentuate these obstacles, thereby reducing opportunities to socialize with peers and to engage in adapted physical activities. These lost opportunities can further generate a vicious cycle of functional decline. Traditional, face-to-face rehabilitation interventions aim to reduce the impact of sensorimotor impairments and provide a means to circumvent these obstacles and assuage the effects of deconditioning to some extent, with some interventions being more effective than others (28). However, given that accessibility to face-to-face programs is greatly limited by the global pandemic, there is an urgent need for a creative solution to the interrupted services, but with full recognition that deferral of the rehabilitation specialists' role to that of a parent is not a desirable solution.

Recently, an expert group of clinicians, researchers and outpatient health program leaders proposed telerehabilitation as a promising strategy to maintain rehabilitation services during this unprecedented time (5). Telerehabilitation is defined as the provision of rehabilitation services via telemedicine methods and techniques (29). This is an ideal strategy to address health issues in low and middle-income countries and remote areas with perpetual restricted access to rehabilitation services (30, 31). This will not only benefit the children with physical disabilities to preserve social contact with their therapist, and maintain improvements already realized, but it could simultaneously reduce the burden on parents. However, telerehabilitation also presents several challenges including: limited training, knowledge and technology/equipment needed for both clinicians and parents as well as a structured therapy intervention (5). Telerehabilitation could be used to facilitate the delivery of rehabilitation services remotely, and the inclusion of active video games and/or low-cost virtual reality, that uses either a synchronous or asynchronous format, in collaboration with a therapist could provide a unique and engaging substitute for in-person services in the current global pandemic.

## Active Video Games and Low-Cost Virtual Reality: A Potential Solution for In-Home Rehabilitation

Active video games and low-cost virtual reality have the potential to engage school-age children and adolescents afflicted by physical disabilities to be physically active while sheltered at home and to continue their engagement with rehabilitation using technology-enhanced game-like interventions. Video games has a large penetration rate in the general population, with over 70% of U.S. families including a child who plays video games (32). Virtual reality, often delivered through low-cost systems, and active video games have increasingly been adopted in rehabilitation practices to mitigate sensorimotor impairments especially for children with CP (28, 33–35). The range of virtual reality technology used for rehabilitation purposes is wide and encompasses video games available commercially to custom-made virtual reality applications specifically designed for rehabilitation applications and with varying degrees of immersion. To demonstrate how rehabilitation services can be offered at home using a virtual reality platform, we focus on systems and applications easily adapted to the home context, including commercially available active video games and low-cost virtual reality applications (AVG/VR). We acknowledge that expensive and research-only virtual reality system may not be a suitable option to reach a large population of children with physical disabilities.

### How Can Active Video Games and Low-Cost Virtual Reality Applications Drive Retention of Motor Skills?

AVG/VR, grounded in principles of experience-dependent neural plasticity and motor learning, offers many advantages to deliver rehabilitation interventions at home. These include: intrinsic motivation, task salience, number of repetitions, intensity and duration, and challenging practice along with the provision of augmented feedback (36). Amidst the current confinement, AVG/VR enables a unique opportunity for skills practice that goes beyond simple household activities; these are engaging skills such as running on the beach, sword fighting or various outdoor sports. A recent study showed that children with physical disabilities are most likely to demonstrate improvements when participating in interventions that are structured, motivating and incorporate various recreation activities (37). Gamification elements, known to drive interest and engagement, are important features of AVG/VR. These very features are more likely to encourage children to be physically active, maintain attention and actively participate in play that is disguised as a rehabilitation program, performed at home and without the formal structure imposed by the rehabilitation setting itself (38–40). Empirical evidence supports progressive practice, that is engaging for the learner and optimally adapted to the individual's capability and the environmental context (41–43). AVG/VR provides the opportunity to customize progressions in task difficulty by incorporating spatial and temporal constraints (40) and to encourage sufficient movement repetitions to drive positive experience-dependent neuroplastic changes (36, 44, 45). Furthermore, meaningful performance feedback can be provided in the form of augmented information about the outcome of the movement and/or the elements of motor performance (17). Feedback type and delivery schedule can be manipulated with AVG/VR. Unlike most tasks performed in the real-world, feedback can be amplified to highlight components of movement performance and quality (44). The numerous benefits of AVG/VR can help clinicians to offer motivating and challenging in-home rehabilitation interventions using a technology often familiar to children and their families.

**Figure 1:**
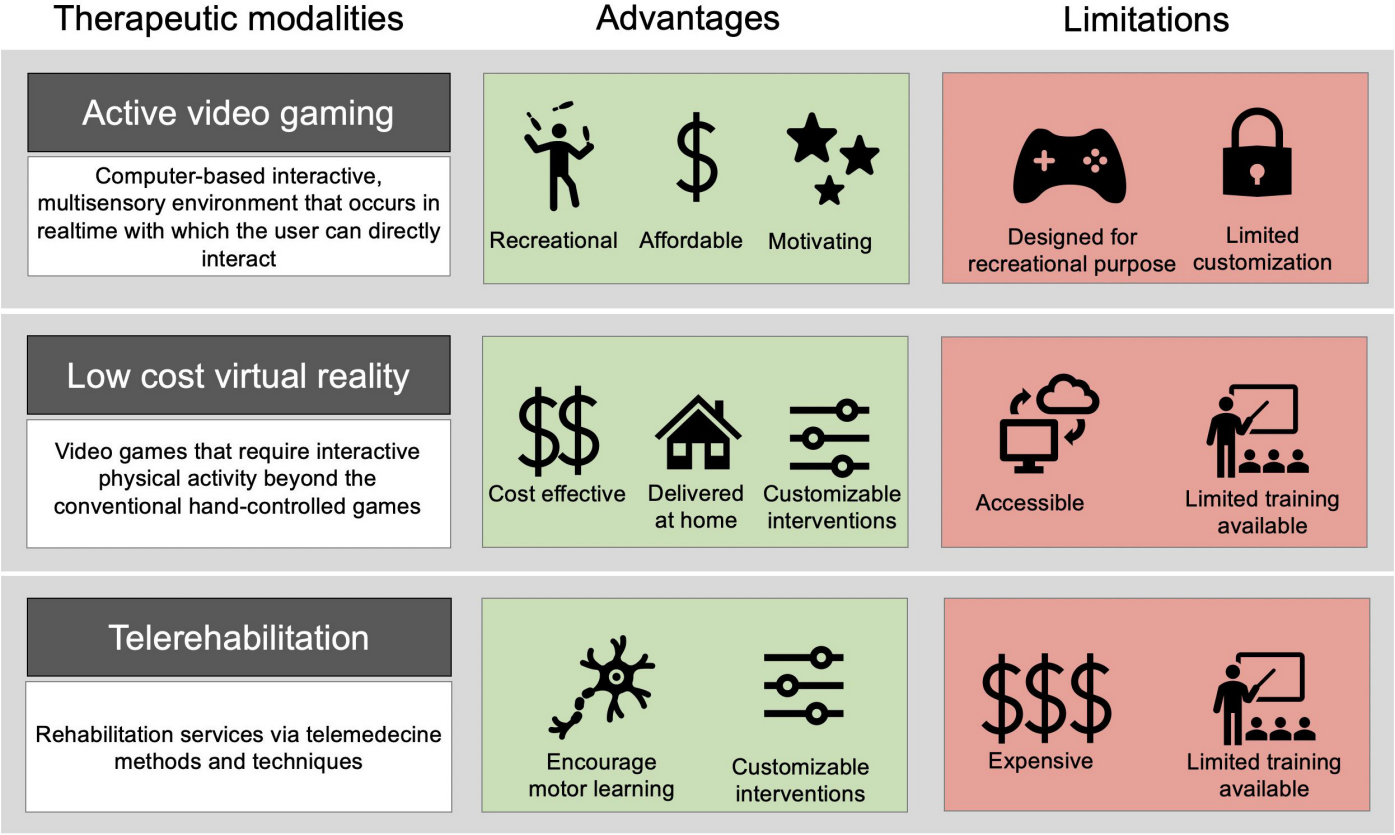
Advantages and limitations of various therapeutic modalities. From left to right: Definition of the therapeutic modalities; list of advantages; list of disadvantages. The combination of AVG/VR with telerehabilitation can be useful to target motor learning principles, such as task-specificity and motivation, to ultimately individualized interventions delivered at home and minimize limitations. We acknowledge that combining these 3 therapeutic modalities can still present limitations, such as limited training for clinicians. Resources such as the Classification Framework by Gavin and Levac (46) can help to address common limitations.

Despite the advantages of AVG/VR for rehabilitation, commercially available active video games are primarily designed for the general population and have limitations that should be acknowledged (Figure 1). Foremost, despite the popularity of these commercial video games, some consoles were discontinued (i.e., Wii™ and Kinect) by gaming companies over the last few years. While the consequences for clinicians remains unclear, this may have an important impact on the sustainability of this technology in rehabilitation (47). Nonetheless, the large penetration rate of commercial video games consoles such as the Wii™ facilitates its accessibility. Another limitation is that many active video games do not all offer sufficient control over the difficulty progression and the task difficulty level may not be suitable for children with more severe motor impairments (48). This stresses the importance of rehabilitation specialists, such as occupational and physical therapists, in the selection of appropriate platforms to best accomplish the individualized treatment goals and for implementing an appropriate level of progression in task difficulty and specification of constraints. When incorporated into a comprehensive treatment plan that includes individualized goals, interventions using AVG/VR can promote the consolidation and retention of motor skills acquired through in-person therapy and in so doing, prevent functional decline mediated by the interruption of vital therapy services.

### Evidence of the Effectiveness of Active Video Games and Low-Cost Virtual Reality in Children With Physical Disabilities

There is growing evidence that supports the use of AVG/VR in children with physical disabilities to achieve improvements across the domains of the International Classification of Functioning, with the bulk of evidence coming from studies of children with CP (28, 33, 40, 46, 49–52). Several studies report the possibility to attain light to moderate levels of physical activity while playing various active video games such as boxing and dancing (53, 54). Active video game practice has been shown to improve voluntary weight shift control while standing for children with CP during a skiing game and standing balance following a short intervention (55, 56). Some preliminary evidence supports the use of AVG/VR to improve upper limb function in children with CP as demonstrated by either kinematic analyses (54, 57) or clinical measures (46, 49, 58–60). Lastly, interventions using AVG/VR can facilitate the transfer of motor skills to the real-life situation, as evidenced by completion of activities of daily living (54, 61, 62). AVG/VR has potential to preserve prior functional improvement and prevent an inevitable decline that will likely occur after a prolonged period in which physical activity is limited (63). While the results are encouraging, the level of evidence is still limited and cannot be generalized across conditions and to all severity of physical disabilities. Moreover, these results were done in a controlled environment with supervision of a clinician ensuring a high adherence and motivation. To reduce the impact of these limitations, the combination of telerehabilitation and AVG/VR, which may facilitate improvements beyond simple maintenance by providing a means to deliver challenging and motivating task-oriented practice and allow the clinician to track progress. To date, many studies from different research groups demonstrate the feasibility to deliver in-home rehabilitation using AVG/VR (either with or without telerehabilitation) for children and adolescents with physical disabilities (64–68). These studies, along with the increasing evidence to support the use of AVG/VR for pediatric physical rehabilitation highlight the potential that this emergent technology has if delivered in participants' home to improve task outcomes and motor function in the current global pandemic.

## Discussion and Recommendations for Clinical Practice

More than ever, children with physical disabilities whose accessibility to rehabilitation services is limited, are more likely to adopt sedentary behaviors (69). Sedentary behavior, physical inactivity and health deconditioning are also likely to be exacerbated due to the confinement and school closures. We propose to leverage AVG/VR to deliver in-home rehabilitation to: (1) minimize the impact of limited rehabilitation services, (2) encourage children with physical disabilities to be physically active within their home environment, and (3) maintain a level of function during this unprecedented global pandemic. With a growing evidence-base in support if AVG/VR in children with physical disabilities, in-home rehabilitation using AVG/VR offers new opportunities to integrate key principles of experience-dependent neuroplasticity and motor learning in the home environment to drive retention of motor skills (i.e., intrinsic motivation, task salience, number of repetitions, intensive and challenging practice, provision of augmented feedback).

Clinicians play a crucial role in selection of an appropriate platform and games to meet individual rehabilitation needs and goals. Clinicians can work with parents and children to explore suitability of various games and consoles already available to the home environment. While frequent follow-up may not be possible, clinicians can offer guidelines on intensity of physical activity, the nature of movement, amount and frequency of active movement and adjustment to the therapeutic goals for each child (70). Results from a recent cross-sectional survey suggests that only ~50% of clinicians have clinical experience using active video games or virtual reality, with lack of knowledge about virtual reality systems, and time to implement them into practice, identified as important barriers to address (71). Resources and knowledge tools can be used to support clinical decision-making about AVG/VR and facilitate the integration into clinical practice. Among already available resources, the “Kinecting with Clinicians” resource (72) and the Nintendo Wii^TM^ game analysis (73) can be used for clinicians to weight the pros and cons of two commercially available active video games. It is also important to note that while the Wii™ and the Kinect are discontinued, other platforms and devices, such as the PlayStation®VR and SteamVR platforms, are currently available, which offer a wide range of active video games that could also be used for rehabilitation purposes. The evolution in AVG/VR technology is rapid and progresses faster than the evidence, which remains a challenge for clinical practice. Two frameworks could help to guide clinical decision-making in gaming choices for therapeutic use: the Classification Framework of pediatric virtual reality systems (46), and the systematic framework (74). As an example, we use the Classification Framework (50) to illustrate how frameworks can be used to guide clinical practice, once AVG/VR systems available in the home environment have been identified. Clinicians can use this framework to identify and select systems and games that can provide multisensory feedback, the opportunity to perform 3D movement to interact with the virtual environment and consistent practice repetitions. As a second step, clinicians can use the seven categories of the framework, such as the ability to manipulate and measure therapeutically relevant variables or the specific movement capacities required, to quickly identify which system meets the needs of the child they are working with or of their current settings. We encourage clinicians to become familiar with AVG/VR prior to adopt it with a child to minimize technical difficulties. When available, the use of clinical champions can also help to foster the development of knowledge and skills of clinicians and facilitate implementation of AVG/VR (75). The choice of platforms and systems often relies of the game consoles available at home. Nonetheless, games incorporating physical activity available on popular gaming consoles (e.g., Nintendo Wii™, PlayStation®VR) should be prioritized since they are user-friendly, interactice and were developed specifically to entice children and adolescents for continuous usage. Guidelines about telerehabilitation from professional associations can be valuable resources help address common barriers to telerehabilitation and facilitate the delivery of remote rehabilitation services (76, 77). To better support clinicians who wish to integrate VR into their practice, our ongoing work includes a systematic review to describe different platforms and active video games in relation to the principles of motor learning (Prospero registration: CRD42020151982).

The past few months taught us that people around the world can develop creative solutions to adapt to the COVID-19 pandemic. The use of AVG/VR could be one creative and evidence-based solution to guide clinicians in the delivery of challenging and motivating in-home physical rehabilitation activities to drive a positive impact on the lives of children with physical disabilities.

## Data Availability Statement

The original contributions generated for the study are included in the article/supplementary material, further inquiries can be directed to the corresponding author.

## Author Contributions

MD and MR conceived the presented idea. MD, OM, and MR critically reviewed the literature. All authors discussed the results and contributed to the final manuscript.

## Conflict of Interest

The authors declare that the research was conducted in the absence of any commercial or financial relationships that could be construed as a potential conflict of interest.

## References

[1] UNESCO Education: From Disruption to Rrecovery. (2020). Available online at: https://en.unesco.org/covid19/educationresponse (accessed August 20, 2020).

[2] Holmes EA O'Connor RC Perry VH Tracey I Wessely S Arseneault L. Multidisciplinary research priorities for the COVID-19 pandemic: a call for action for mental health science. Lancet Psychiatry. (2020) 7:547–560. 10.1016/S2215-0366(20)30168-1 32304649 PMC7159850

[3] World Health Organization COVID-19 Strategy Update. (2020). Available online at: www.who.int/publications/i/item/strategic-preparedness-and-response-plan-for-the-new-coronavirus (accessed August 20, 2020).

[4] UNICEF COVID-19 Response: Considerations for Children and Adults with Disabilities. (2020). Available online at: https://www.unicef.org/disabilities/files/COVID-19_response_considerations_for_people_with_disabilities_190320.pdf (accessed August 20, 2020).

[5] Turolla A Rossettini G Viceconti A Palese A Tommaso G. Musculoskeletal physical therapy during the COVID-19 pandemic: is telerehabilitation the answer? Phys Ther. (2020) 100:1260–4. 10.1093/ptj/pzaa131 32386218 PMC7239136

[6] Negrini S Grabljevec K Boldrini P Kiekens C Moslavac S Zampolini M. Up to 2.2 million people experiencing disability suffer collateral damage each day of Covid-19 lockdown in Europe. Eur J Phys Rehabil Med. (2020) 56:361–5. 10.23736/S1973-9087.20.06361-3 32383576

[7] Winstein CJ Requejo PS Zelinski EM Mulroy SJ Crimmins EM. A transformative subfield in rehabilitation science at the nexus of new technologies, aging, and disability. Front Psychol. (2012) 3:1–8. 10.3389/fpsyg.2012.00340 23049517 PMC3448347

[8] Mears D Hansen L Active gaming: definitions, options and implementation. Article# 5 in a 6-Part Series. Strategies. (2009) 23:26–9. 10.1080/08924562.2009.10590864

[9] Adamovich SV Fluet GG Tunik E Merians AS. Sensorimotor training in virtual reality: a review. NeuroRehabilitation. (2009) 25:29–44. 10.3233/NRE-2009-0497 19713617 PMC2819065

[10] Bishop DVM. Which neurodevelopmental disorders get researched and why? PLoS ONE. (2010) 5:e15112. 10.1371/journal.pone.0015112 21152085 PMC2994844

[11] Paneth N Hong T Korzeniewski S. The descriptive epidemiology of cerebral palsy. Clin Perinatol. (2006) 33:251–67. 10.1016/j.clp.2006.03.011 16765723

[12] Shevell M Dagenais L Oskoui M. The epidemiology of cerebral palsy: new perspectives from a Canadian registry. Semin Pediatr Neurol. (2013) 20:60–4. 10.1016/j.spen.2013.06.008 23948680

[13] Oskoui M Coutinho F Dykeman J Jetté N Pringsheim T. An update on the prevalence of cerebral palsy: a systematic review and meta-analysis. Dev Med Child Neurol. (2013) 55:509–19. 10.1111/dmcn.12080 23346889

[14] Katz RT Johnson CB. Life care planning for the child with cerebral palsy. Phys Med Rehabil Clin N Am. (2013) 24:491–505. 10.1016/j.pmr.2013.03.003 23910487

[15] Shortland A. Muscle deficits in cerebral palsy and early loss of mobility: can we learn something from our elders? Dev Med Child Neurol. (2009) 51(Suppl. 4):59–63. 10.1111/j.1469-8749.2009.03434.x 19740211

[16] Auld ML Boyd RN Moseley GL Ware RS Johnston LM. Impact of tactile dysfunction on upper-limb motor performance in children with unilateral cerebral palsy. Arch Phys Med Rehabil. (2012) 93:696–702. 10.1016/j.apmr.2011.10.025 22360974

[17] Robert MT Sambasivan K Levin MF. Extrinsic feedback and upper limb motor skill learning in typically-developing children and children with cerebral palsy: review. Restor Neurol Neurosci. (2017) 35:171–84. 10.3233/RNN-160688 28282845

[18] Verschuren O Wiart L Hermans D Ketelaar M. Identification of facilitators and barriers to physical activity in children and adolescents with cerebral palsy. J Pediatr. (2012) 161:488–94. 10.1016/j.jpeds.2012.02.042 22494875

[19] Bodimeade HL Whittingham K Lloyd O Boyd RN. Executive function in children and adolescents with unilateral cerebral palsy. Dev Med Child Neurol. (2013) 55:926–33. 10.1111/dmcn.12195 23809003

[20] Straub K Obrzut JE Effects of cerebral palsy on neuropsychological function. J Dev Phys Disabil. (2009) 21:153–67. 10.1007/s10882-009-9130-3

[21] Law M King G King S Kertoy M Hurley P Rosenbaum P. Patterns of participation in recreational and leisure activities among children with complex physical disabilities. Dev Med Child Neurol. (2006) 48:337–42. 10.1017/S0012162206000740 16608540

[22] Brown M Gorden WA. Impact of impairment on activity patterns of children. Arch Phys Med Rehabil. (1987) 68:828–32. 2962558

[23] Shikako-Thomas K Majnemer A Law M Lach L. Determinants of participation in leisure activities in children and youth with cerebral palsy: systematic review. Phys Occup Ther Pediatr. (2008) 28:155–69. 10.1080/01942630802031834 18846895

[24] Anaby D Hand C Bradley L Direzze B Forhan M Digiacomo A. The effect of the environment on participation of children and youth with disabilities: a scoping review. Disabil Rehabil. (2013) 35:1589–98. 10.3109/09638288.2012.748840 23350759

[25] Anaby D Law M Coster W Bedell G Khetani M Avery L. The mediating role of the environment in explaining participation of children and youth with and without disabilities across home, school, and community. Arch Phys Med Rehabil. (2014) 95:908–17. 10.1016/j.apmr.2014.01.005 24468018

[26] Law M Anaby D Teplicky R Khetani MA Coster W Bedell G Participation in the home environment among children and youth with and without disabilities. Br J Occup Ther. (2013) 76:58–66. 10.4276/030802213X13603244419112

[27] Shikako-Thomas K Shevell M Schmitz N Lach L Law M Poulin C. Determinants of participation in leisure activities among adolescents with cerebral palsy. Res Dev Disabil. (2013) 34:2621–34. 10.1016/j.ridd.2013.05.013 23751302

[28] Novak I Morgan C Fahey M Finch-Edmondson M Galea C Hines A. State of the evidence traffic lights 2019: systematic review of interventions for preventing and treating children with cerebral palsy. Curr Neurol Neurosci Rep. (2020) 20:3. 10.1007/s11910-020-1022-z 32086598 PMC7035308

[29] Brennan DM Tindall L Theodoros D Brown J Campbell M Christiana D. A blueprint for telerehabilitation guidelines–October 2010. Telemed J E Health. (2011) 17:662–5. 10.1089/tmj.2011.0036 21790271

[30] Winkler AS. The growing burden of neurological disorders in low-income and middle-income countries: priorities for policy making. Lancet Neurol. (2020) 19:200–2. 10.1016/S1474-4422(19)30476-4 31813849

[31] Longo E de Campos AC Schiariti V. COVID-19 pandemic: is this a good time for implementation of home programs for children's rehabilitation in low- and middle-income countries? Phys Occup Ther Pediatr. (2020) 40:361–4. 10.1080/01942638.2020.1759947 32408834

[32] Entertainment Software Association Essential Facts About the Computer and Video Game Industry 2019. (2019). Available online at: www.theesa.com/esa-research/2019-essential-facts-about-the-computer-and-video-game-industry/ (accessed August 20, 2020).

[33] Johansen T Strøm V Simic J Rike PO. Effectiveness of training with motion-controlled commercial video games for hand and arm function in people with cerebral palsy: a systematic review and meta-analysis. J Rehabil Med. (2020) 52:jrm00012. 10.2340/16501977-2633 31794044

[34] Levac DE Galvin J. When is virtual reality “therapy”? Arch Phys Med Rehabil. (2013) 94:795–8. 10.1016/j.apmr.2012.10.021 23124132

[35] Weiss PL Tirosh E Fehlings D. Role of virtual reality for cerebral palsy management. J Child Neurol. (2014) 29:1119–24. 10.1177/0883073814533007 24799367

[36] Kleim JA Jones TA. Principles of experience-dependent neural plasticity: implications for rehabilitation after brain damage. J Speech Lang Hear Res. (2008) 51:225–39. 10.1044/1092-4388(2008/018) 18230848

[37] McCoy SW Palisano R Avery L Jeffries L Laforme Fiss A Chiarello L. Physical, occupational, and speech therapy for children with cerebral palsy. Dev Med Child Neurol. (2020) 62:140–6. 10.1111/dmcn.14325 31353456

[38] Wang M Reid D. Virtual reality in pediatric neurorehabilitation: attention deficit hyperactivity disorder, autism and cerebral palsy. Neuroepidemiology. (2011) 36:2–18. 10.1159/000320847 21088430

[39] Liebert MA Bryanton C Pt BS Bossé J Pt BS Brien M. Feasibility, motivation, and selective motor control: virtual reality compared to conventional home exercise in children with cerebral palsy. Cyberpsychol Behav. (2006) 9:123–8. 10.1089/cpb.2006.9.123 16640463

[40] Snider L Majnemer A Darsaklis V. Virtual reality as a therapeutic modality for children with cerebral palsy. Dev Neurorehabil. (2010) 13:120–8. 10.3109/17518420903357753 20222773

[41] Guadagnoli MA Lee TD. Challenge point: a framework for conceptualizing the effects of various practice conditions in motor learning. J Mot Behav. (2004) 36:212–24. 10.3200/JMBR.36.2.212-224 15130871

[42] Plautz EJ Milliken GW Nudo RJ. Effects of repetitive motor training on movement representations in adult squirrel monkeys: role of use versus learning. Neurobiol Learn Mem. (2000) 74:27–55. 10.1006/nlme.1999.3934 10873519

[43] Winstein CJ Kay DB. Translating the science into practice. Prog Brain Res. (2015) 218:331–60. 10.1016/bs.pbr.2015.01.004 25890145

[44] Levin MF Demers M Motor learning in neurological rehabilitation. Disabil Rehabil. (2020) 1–9. 10.1080/09638288.2020.1752317. [Epub ahead of print]. 32320305

[45] Crosbie JH Lennon S Basford JR McDonough SM. Virtual reality in stroke rehabilitation: still more virtual than real. Disabil Rehabil. (2007) 29:1139–46. 10.1080/09638280600960909 17613000

[46] Galvin J Levac D. Facilitating clinical decision-making about the use of virtual reality within paediatric motor rehabilitation: describing and classifying virtual reality systems. Dev Neurorehabil. (2011) 14:112–22. 10.3109/17518423.2010.535805 21410403

[47] Bonnechère B Omelina L Kostkova K Van Sint Jan S Jansen B. The end of active video games and the consequences for rehabilitation. Physiother Res Int. (2018) 23:1–2. 10.1002/pri.1752 30259613

[48] Demers M Fung K Subramanian SK Lemay M Robert MT Incorporation of motor learning principles in virtual rehabilitation in individuals with cerebral palsy: a systematic review. JMIR Serious Games Prepr [Preprint]. (2020). 10.2196/23822 PMC8060861 33825690

[49] Chen YP Lee SY Howard AM. Effect of virtual reality on upper extremity function in children with cerebral palsy: a meta-analysis. Pediatr Phys Ther. (2014) 26:289–300. 10.1097/PEP.0000000000000046 24819682

[50] Hickman R Popescu L Manzanares R Morris B Lee SP Dufek JS. Use of active video gaming in children with neuromotor dysfunction: a systematic review. Dev Med Child Neurol. (2017) 59:903–11. 10.1111/dmcn.13464 28542867

[51] Massetti T Silva TD da Ribeiro DC Malheiros SRP Ré AHN Favero FM Motor learning through virtual reality in cerebral palsy—a literature review. Med Express. (2014) 1:302–6. 10.5935/MedicalExpress.2014.06.04

[52] Mitchell L Ziviani J Oftedal S Boyd R. The effect of virtual reality interventions on physical activity in children and adolescents with early brain injuries including cerebral palsy. Dev Med Child Neurol. (2012) 54:667–71. 10.1111/j.1469-8749.2011.04199.x 22283557

[53] Howcroft J Klejman S Fehlings D Wright V Zabjek K Andrysek J. Active video game play in children with cerebral palsy: potential for physical activity promotion and rehabilitation therapies. Arch Phys Med Rehabil. (2012) 93:1448–56. 10.1016/j.apmr.2012.02.033 22571917

[54] Robert MT Guberek R Sveistrup H Levin MF. Motor learning in children with hemiplegic cerebral palsy and the role of sensation in short-term motor training of goal-directed reaching. Dev Med Child Neurol. (2013) 55:1121–8. 10.1111/dmcn.12219 23899048

[55] Deutsch JE Borbely M Filler J Huhn K Guarrera-Bowlby P. Use of a low-cost, commercially available gaming console (Wii) for rehabilitation of an adolescent with cerebral palsy. Phys Ther. (2008) 88:1196–207. 10.2522/ptj.20080062 18689607

[56] Ballaz L Robert M Parent A Prince F Lemay M. Impaired visually guided weight-shifting ability in children with cerebral palsy. Res Dev Disabil. (2014) 35:1970–7. 10.1016/j.ridd.2014.04.019 24858794

[57] Uswatte G Foo WL Olmstead H Lopez K Holand A Simms LB. Arm use in patients with subacute stroke monitored by accelerometry: association with motor impairment and influence on self-dependence. Arch Phys Med Rehabil. (2011) 31:16–33. 10.2340/16501977-0676 21347506

[58] Fluet GG Qiu Q Kelly D Parikh HD Ramirez D Saleh S. Interfacing a haptic robotic system with complex virtual environments to treat impaired upper extremity motor function in children with cerebral palsy. Dev Neurorehabil. (2010) 13:335–45. 10.3109/17518423.2010.501362 20828330 PMC3025751

[59] Luna-Oliva L Ortiz-Gutiérrez RM Cano-De La Cuerda R Piédrola RM Alguacil-Diego IM Sánchez-Camarero C Kinect Xbox 360 as a therapeutic modality for children with cerebral palsy in a school environment: a preliminary study. NeuroRehabilitation. (2013) 33:513–21. 10.3233/NRE-131001 24018364

[60] Weightman A Preston N Levesley M Holt R Mon-Williams M Clarke M. Home-BASED computer-assisted upper limb exercise for young children with cerebral palsy: a feasibility study investigating impact on motor control and functional outcome. J Rehabil Med. (2011) 43:359–63. 10.2340/16501977-0679 21347508

[61] de Mello Monteiro CB Massetti T da Silva TD van der Kamp J de Abreu LC Leone C. Transfer of motor learning from virtual to natural environments in individuals with cerebral palsy. Res Dev Disabil. (2014) 35:2430–7. 10.1016/j.ridd.2014.06.006 24981192

[62] Winkels DGM Kottink AIR Temmink RAJ Nijlant JMM Buurke JH. WiiTM-habilitation of upper extremity function in children with cerebral palsy. An explorative study. Dev Neurorehabil. (2013) 16:44–51. 10.3109/17518423.2012.713401 23030054

[63] Bonnechère B Jansen B Omelina L Degelaen M Wermenbol V Rooze M. Can serious games be incorporated with conventional treatment of children with cerebral palsy? A review. Res Dev Disabil. (2014) 35:1899–913. 10.1016/j.ridd.2014.04.016 24794289

[64] Golomb MR McDonald BC Warden SJ Yonkman J Saykin AJ Shirley B. In-home virtual reality videogame telerehabilitation in adolescents with hemiplegic cerebral palsy. Arch Phys Med Rehabil. (2010) 91:1–8.e1. 10.1016/j.apmr.2009.08.153 20103390

[65] Huber M Rabin B Docan C Burdea G Nwosu ME Abdelbaky M PlayStation 3-based tele-rehabilitation for children with hemiplegia. Virtual Rehabil IWVR. (2008) 105–12. 10.1109/ICVR.2008.4625145

[66] Reifenberg G Gabrosek G Tanner K Harpster K Proffitt R Persch A. Feasibility of pediatric game-based neurorehabilitation using telehealth technologies : a case report. Am J Occup Ther. (2017) 71:7103190040p1-7103190040p8. 10.5014/ajot.2017.024976 28422630

[67] Kassee C Hunt C Holmes MWR Lloyd M. Home-based Nintendo Wii training to improve upper-limb function in children ages 7 to 12 with spastic hemiplegic cerebral palsy. J Pediatr Rehabil Med. (2017) 10:145–54. 10.3233/PRM-170439 28582885

[68] Sandlund M Grip H Häger C Domellöf E Ronnqvist L Low-cost motion interactive video games in home training for children with cerebral palsy: a kinematic evaluation. Int Conf Virtual Rehabil ICVR. (2011) 1–2. 10.1109/ICVR.2011.5971854

[69] Buffart LM Westendorp T Van Den Berg-Emons RJ Stam HJ Roebroeck ME. Perceived barriers to and facilitators of physical activity in young adults with childhood-onset physical disabilities. J Rehabil Med. (2009) 41:881–5. 10.2340/16501977-0420 19841838

[70] Deutsch JE Westcott McCoy S. Virtual reality and serious games in neurorehabilitation of children and adults: prevention, plasticity, and participation. Pediatr Phys Ther. (2017) 29:S23-36. 10.1097/PEP.0000000000000387 28654475 PMC5488703

[71] Levac D Glegg S Colquhoun H Miller P Noubary F. Virtual reality and active videogame-based practice, learning needs, and preferences: a cross-Canada survey of physical therapists and occupational therapists. Games Health J. (2017) 6:217–28. 10.1089/g4h.2016.0089 28816511

[72] Levac D Espy D Fox E Pradhan S Deutsch JE. “Kinect-ing” with clinicians: a knowledge translation resource to support decision making about video game use in rehabilitation. Phys Ther. (2015) 95:426–40. 10.2522/ptj.20130618 25256741 PMC4348717

[73] Deutsch JE Brettler A Smith C Welsh J John R Guarrera-Bowlby P. Nintendo Wii sports and Wii fit game analysis, validation, and application to stroke rehabilitation. Top Stroke Rehabil. (2011) 18:701–19. 10.1310/tsr1806-701 22436308

[74] Espy D Reinthal A Dal Bello-Haas V A systematic framework to guide clinical decision-making in gaming choices for therapeutic use. In: APTA Combined Sections Meeting. Salt Lake City, UT (2013).

[75] Glegg SMN Levac DE. Barriers, facilitators and interventions to support virtual reality implementation in rehabilitation: a scoping review. PM&R. (2018) 10:1237–51.e1. 10.1016/j.pmrj.2018.07.004 30503231 PMC6752033

[76] Occupational Therapy of Australia Telehealth. (2020). Available online at: www.otaus.com.au/member-resources/covid-19/telehealth (accessed November 18, 2020).

[77] Charted Society of Physiotherapy Covid-19: Guide for Rapid Implementation of Remote Physiotherapy Delivery. (2020). Available online at: www.csp.org.uk/publications/covid-19-guide-rapid-implementation-remote-physiotherapy-delivery (accessed November 18, 2020).

